# Inhibition of Angiogenesis: A Novel Effect of Zataria Multiflora

**Published:** 2017-04-01

**Authors:** Amir Hossein Norooznezhad, Maryam Keshavarz, Fatemeh Norooznezhad, Kamran Mansouri

**Affiliations:** 1 Medical Biology Research Center, Kermanshah University of Medical Sciences, Kermanshah, Iran; 2 Shariati Hospital, Tehran University of Medical Sciences, Tehran, Iran

**Keywords:** Angiogenesis, Zataria multiflora, Endothelial cells, Cell migration

## Abstract

**Background** **:** Angiogenesis, the formation of new blood vessels from preexisting ones, is among the most important physiological and pathological processes that occur in the body. Under pathological conditions such as tumor growth, psoriasis, corneal neovascularization and rheumatoid arthritis, angiogenesis is substantial for the development of the disease. Zataria multiflora is a member of the Labiatae family with a vast range of traditional uses which has been long known and applied in Iran old medicine. The aim of this study was the evaluation of anti-angiogenic potential of Zataria multiflora.

**Materials and Methods: **In this study, human umbilical vein endothelial cells (HUVECs) were isolated from newborn umbilical veins and then cultured for cytotoxicity (LDH test) assay. Regarding LDH results, following tests such as angiogenesis (cytodex-3 micro carrier) and migration (wound healing) tests were designed.

**Results:** The cytotoxicity assays showed no toxicity of Z.multiflora toward HUVECs in the range of 10-450µg/mL of the extract. This extract was also able to inhibit angiogenesis and migration at 200µg/mL.

**Conclusion:** Our data clearly demonstrated an inhibitory effect of Z. multiflora on angiogenesis and migration of HUVECs. Z. multiflora could be introduced as a significant angiogenesis inhibitor for angiogenesis-dependent diseases in further complementary studies.

## Introduction

 Angiogenesis process is described as the formation of new blood vessels from preexisting ones. The process is affected by some endogenous inducer factors, which are normally in balance with their inhibitors. When this balance is shattered, either induction or inhibition of angiogenesis may occur.^1^ Excessive angiogenesis has been found to incorporate with certain diseases such as corneal neovascularization,^2^^,^^3^psoriasis,^4^ rheumatoid arthritis^5^ and solid tumor growth.^1^Tumor induced angiogenesis, as a result of hypoxia, begins when tumor cells undergoing hyper proliferation. In this situation, angiogenesis helps the tumor cells by providing nutrients, oxygen and aids in the removal of waste. Due to related needs, the balance between angiogenesis inhibitors and inducers must be changed in favor of angiogenesis inducers.^6^ This process starts with degradation of basement membrane and proliferation, migration and tube formation of vascular endothelial cells.^7^ Each step in angiogenesis is affected by various factors such as hypoxia inducible factor-1 (HIF-1), vascular endothelial growth factor (VEGF), basic fibroblast growth factor (bFGF), matrix metalloproteinases (MMPs), platelet-derived growth factor (PDGF), etc.^8^ Nowadays, angiogenesis-related therapy is among the most notable strategies for the treatment of angiogenesis-dependent disease, especially in the case of solid tumors.^1^ In 2008, more than 1.2 million patients were being treated with anti-angiogenic agents.^9^

Zatariamultiflora is a well-known plant with many benefits in limited geographical cultivation area including Iran and Afghanistan. Besides being traditionally applied for its anti-spasmodic and anti-septic activity,^10^ this plant which goes by the local name of Avishan-e-Shirazi is also used in some Iranian foods.^11^During recent years, some investigations have been carried out on medical advantages of this native plant including anti-inflammatory, antinociceptive,^12^^,^^13^ antimicrobial and antioxidant activity,^14^ etc.

The aim of the present study was to evaluate the anti-angiogenic activity of this valuable plant in an in vitro model of angiogenesis.

## MATERIALS AND METHODS

**Plant Material and Extraction**

Plant samples were collected in July 2013 in Shiraz. Samples were washed, air-dried and then milled to a fine powder. This powder was then blended in deionized water with the ratio of 1/4 (W/V) for 24 hours in 4ºC. Afterwards, the mixture was centrifuged in 4000×g for 10 minutes. Finally, supernatant was collected and dried at 37 ºC for further uses.

**Cell Isolation and Culture**

Desired cells were isolated from human umbilical vein of newborns according to Mostafaie et al.’s method using actinidin enzyme as a collagenase.^15^ Cells were then cultured in Dulbecco’s Modified Eagle Medium (DMEM) containing 10% Fetal Bovine Serum (FBS) at 37ºC and 5% CO2.

**Cytotoxicity Assay**

Cytotoxic concentrations of Zataria M extract which reduced the viability of HUVECs by 50% were determined in this test. The cells were treated with different concentrations (10-700 µg/mL) of the extract. After 48h of incubation at 37°c and 5% CO2, cell viability was determined through trypan blue exclusion and lactate dehydrogenase (LDH) assays. The absorbance of converted dye in LDH assay was measured at over wavelength of 490 nm with background subtraction at 630 nm.^16^

**Angiogenesis Assay**

HUVECs were cultured in MCDB131 medium supplemented with 10% FBS at 37 ºC and 5% CO2. To create an in vitro system of angiogenesis, the cells were cultured in a standard microcarrier-based model in collagen gel.^17^^,^^18^ After mixing cells with cytodex 3- microcarriers and shaking every 20 minutes for 4h, the mixture was finally transferred to a 24-well tissue culture plate and rested for 12-16 h in MCDB131 supplemented with 10% FBS at 37 ºC and 5% CO2. After 24 h, cells attached to the beads were resuspended in type-1 collagen gel, and the final mixture was divided into a 96-well tissue culture plate. Then, MCDB131 medium was added to each well and after 8-12h different doses of the extract was added. After 3-5 days of treatment, anti-angiogenic effects of the extract were monitored microscopically.

**Migration Assay**

**Scratch Motility (Wound Healing) Assay**

The scratch assay is a useful and a straightforward method for evaluation of cell migration in vitro. Control and treated groups at the same cell number were seeded into 24-well plates. The next step involved creating a cell-free area of confluent monolayer cells by scraping the cell monolayer with a 100μl pipette tip on the following day. After washing the detached cells, images were captured during a 24-h period of cell migration.

For comparing the migration rate, scratches were photographed at five separate fields and then free cell area was analyzed by NIH Image J software. The percentage of wound closure (inhibition %) was estimated as previously described by the following equation: wound closure%= [1−(wound area at t1/wound area at t0) × 100%], where t1 is the time after 24 h of wounding and t0 is the time immediately after wounding (t0 is just after wound was made and t1 is after 24h of treating with extract).^19^

**Proliferation Assay**

HUVECs were cultured in MCDB131 medium supplemented with 10% FBS at 37 ºC and 5% CO2 in a 96-well tissue culture plate. Different doses of the extract were then added and cell counting was carried out using a culture counter (KX-21, SysmexCo). After three days, the obtained results were then compared to controls which were not treated with extract.

**Statistical Analysis**

The obtained data were analyzed using GraphPad Prism® software as well as graph designing.

## Results

**Cell Isolation**

Collagenase activity of actinidin enzyme in isolating cells from certain tissues has already been confirmed by Mostafaie al.^14^ HUVECs were isolated from the vein of newborns using this method. The cells obtained were able to proliferate in cell culture situation and experimental conditions for angiogenesis and migration assay as described in methods.

**Cytotoxicity**

According to the LDH test, the extract was not cytotoxic at doses lower than 450µg/mL. This result has been confirmed by trypan assay as well as LDH method result.

**Figure 1:**
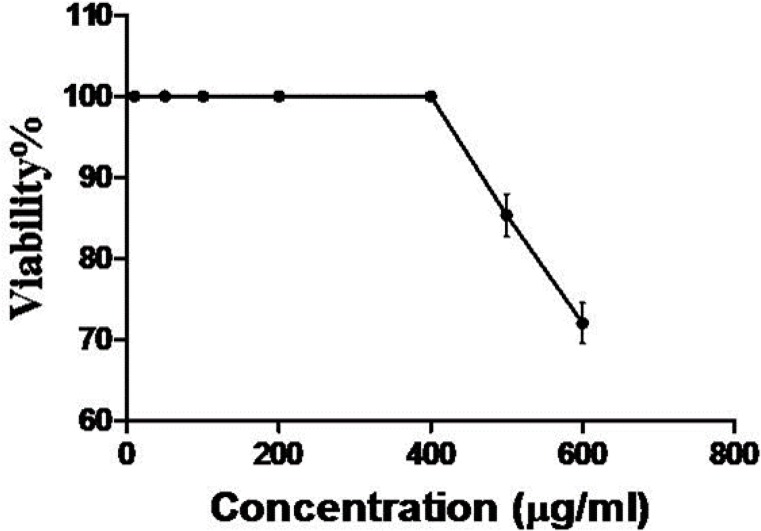
Evaluation of cytotoxic activity of extract on HUVECs using LDH and trypan blue assay

**Inhibition of Angiogenesis**

**Figure 2:**
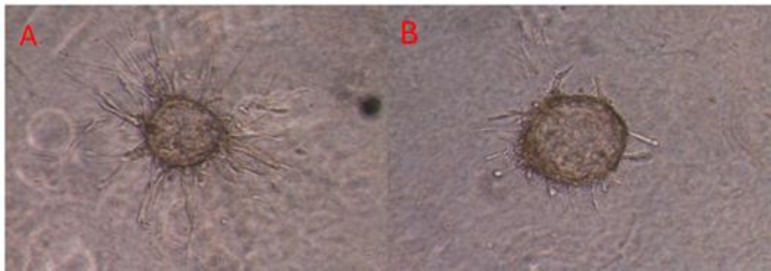
Cytodex 3- microcarriers in collagen gel A: Control. B: 200µg/mL of extract which completely prevented angiogenesis.

**Figure 3:**
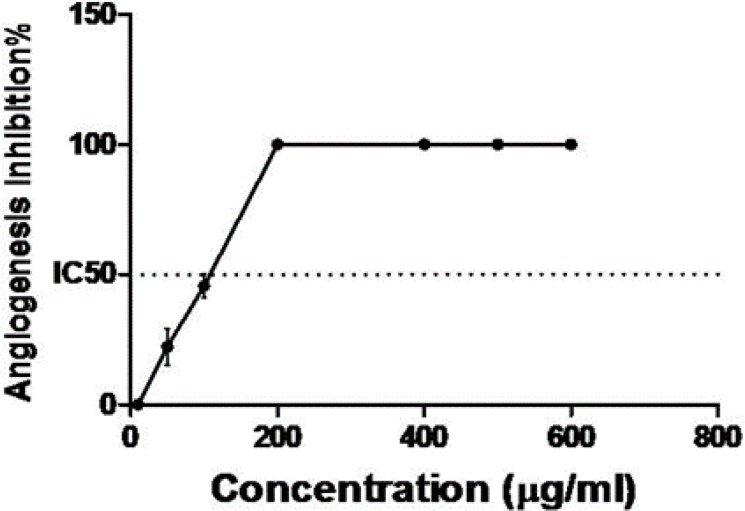
Inhibition of angiogenesis by Zataria M extract

Our results clearly established the anti-angiogenic activity of Zataria M. This medicinal plant was able to inhibit angiogenesis in vitro at doses lower than the toxic dose (Figure 1). Starting point for inhibition affirmed 200µg/mL (Figure 2) of the extract which completely prevented angiogenesis process (100% inhibition, Figure 3).

**Migration Assay**

**Figure 4:**
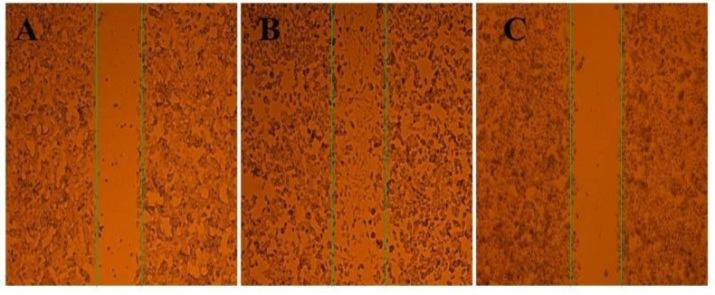
Migration assay of HUVECs using wound healing model. A) HUVECs right after wound formation, B) Control after 24 h, wounded cell monolayers of HUVEC completely filled in the cleared area, C) 200µg/mL of extract which completely inhibited migration of HUVECs.

**Figure 5:**
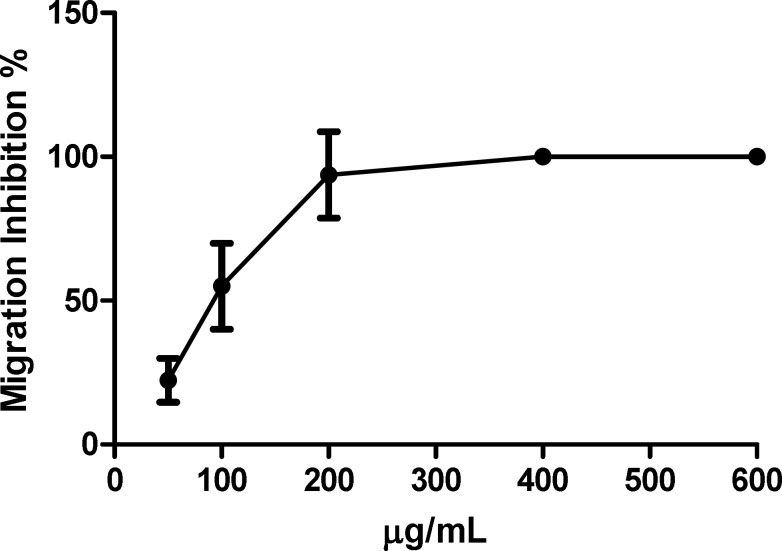
Inhibition of HUVECs’ migration at 200 µg/mL motility was significantly hindered.

Migration is of the most important steps of angiogenesis that was also investigated in the present study. Wound-healing assays were used to determine the ability of Zataria M extract for inhibition of HUVECs migration. After 24 h, wounded monolayer of HUVECs (non-treated cells) was completely filled in the cleared scratched area (control), while in those treated with different concentrations of extract migration ratio was significantly suppressed. As shown in Figure 4, the extract was able to inhibit the migration of HUVECs in this method. Interestingly, treatment with 200 µg/mL of extract significantly decreased EC’s migration ratio which was observed in other similar concentrations (data are shown in Figure 5 by % of inhibition).

**Proliferation Assay**

**Figure 6:**
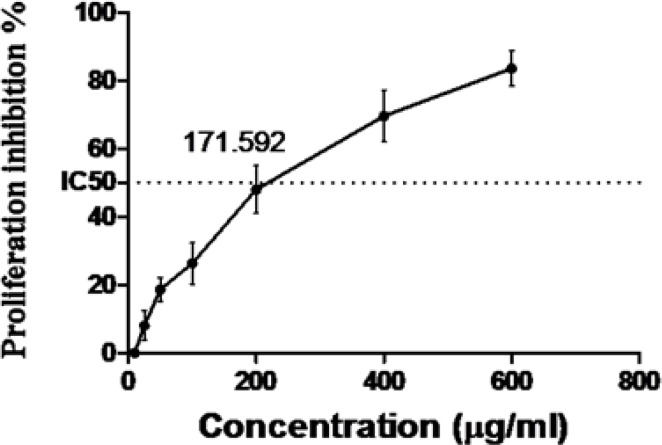
Inhibition of HUVECs proliferation by Zataria M extract

After counting HUVECs using a cell counter, the data showed that the extract was able to inhibit proliferation in a dose-dependent manner. Proliferation was suppressed more than 70% at 400µg/mL which is lower than the toxic dose. Furthermore, IC50% was calculated 171.5µg/mL which is less than the complete angiogenic inhibitory dose (Figure 6).

Different concentrations of extract (50, 75, 100, 150, 200, 400 and 600µg/mL) were used in the mentioned tests.

## Discussion

 Recently, more attention has been paid to the anti-angiogenic agents with different resources ^20^^,^^21^ such as protein: Kunitz trypsin inhibitor^22^ or shark cartilage fractions,^23^ plant materials: Allium ascalonicum,^24^ salvia officinalis^25^ and FicusCarica’s latex,^26^ Cannabinoids,^27^ even mushrooms^28^ or monoclonal antibodies such as Bevacizumab.^6^Although all these agents are able to inhibit angiogenesis, plant-derived substances seem more cost beneficial, highly available and also resistant to proteinases when used orally.As mentioned before, migration plays an important role in angiogenesis and allows the proliferated endothelial cells to migrate to the selected area.^7^Zatariamultiflora is one of the traditional plants in Iran which was used as an anti-angiogenic agent in vitro in this study. It was also shown that Zataria multiflora is able to inhibit angiogenesis in a dose- dependent manner. Toxic effects appeared in dose of 450µg/mL and concentrations less than this value showed angiogenic inhibitory effects. The extract suppressed ECs proliferation in an acceptable dose, which means it could depress an important step of angiogenesis.^7^As reported, salvia officinalis extract has anti-angiogenic potential which could inhibit the proliferation like Zataria multiflora.

In a study by Keshavarz et al., salvia officinalis extract significantly suppressed ECs proliferation in 300µg/mL. The same result was also obtained in this study with the use of Zataria multiflora. Our extract was also able to inhibit migration of HUVECs, which is one of the most important steps in tumor angiogenesis. Zataria multiflora significantly inhibited the ECs migration in 200µg/mL, while salvia officinalis did it in 300µg/mL. Moreover, Allium ascalonicum, anti-angiogenic extract, could inhibit tube formation, migration and proliferation of ECs in and 300, respectively. Also, Allium hirtifolium, anti-angiogenic plant extract, could suppress ECs’ tube formation (500µg/mL), proliferation (300µg/mL for IC50) and migration (500µg/mL).^29^It seems that Zataria multiflora is more potent than Allium hirtifolium in plant extract category of anti-angiogenic agents. Upon inhibiting migration in tumoral tissues, proliferated HUVECs would not be able to migrate to the wounded area. It seems that Zataria multiflora, due to its potential on inhibition of angiogenesis proliferation and migration of HUVECs, may have to be a possible candidate for future anti-angiogenic agents. 

## CONCLUSION

 All together, we suggest other complementary studies which can be carried out on the molecular basis of angiogenesis inhibition of Zataria M to study the possible effect of this plant on HIF-1, VEGF, MMPs or even purification of active compound(s) of Zataria multiflora.

## References

[1] Mostafaie A, Mohammadi-Motlagh HR, Mansouri K (2010). Angiogenesis and the models to study angiogenesis. Yakhteh Med J.

[2] Norooznezhad AH, Norooznezhad F (2016). How Could Cannabinoids Be Effective in Multiple Evanescent White Dot Syndrome? A Hypothesis. J Rep Pharm Sci.

[3] Shakiba Y, Mansouri K, Arshadi D (2009). Corneal neovascularization: molecular events and therapeutic options. Recent Pat Inflamm Allergy Drug Discov.

[4] Heidenreich R, Röcken M, Ghoreschi K (2008). Angiogenesis: the new potential target for treatment of psoriasis?. Drug News Perspect.

[5] Szekanecz Z, Koch AE (2009). Angiogenesis and its targeting in rheumatoid arthritis. Vascul Pharmacol.

[6] Nguyen A, Hoang V, Laquer V (2009). Angiogenesis in cutaneous disease:Part I. J Am Acad Dermatol.

[7] Plank MJ, Sleeman BD (2004). Tumor–induced angiogenesis: A review. J Theor Med.

[8] Kuwano M, Fukushi J, Okamoto M (2001). Angiogenesis factors. Intern Med.

[9] Bischoff J, Griffioen AW (2008). In memoriam Dr. Judah Folkman. Angiogenesis.

[10] Zargari A (1990). Medicinal Plants.

[11] Jafari S, Amanlou M, Borhan-Mojabi K (2003). Comparative study of Zataria multiflora and Anthemis nobelis extracts with Myrthus communis preparation in the treatment of recurrent aphthous stomatitis. Daru.

[12] Jaffary F, Ghannadi A, Siahpoush A (2004). Antinociceptive effects of hydroalcoholic extract and essential oil of Zataria multiflora. Fitoterapia.

[13] Hosseinzadeh H, Ramezani M, Salmani GA (2000). Antinociceptive, anti inflammatory and acute toxicity effects of Zataria multiflora Boiss extracts in mice and rats. J Ethnopharmacol.

[14] Sharififar F, Moshafi MH, Mansouri SH (2007). In vitro evaluation of antibacterial and antioxidant activities of the essential oil and methanol extract of endemic Zataria multiflora Boiss. Food Control.

[15] Bidmeshkipour A, Shirvani Farsani Z (2007). Isolation of human umbilical vein endothelial cells using kiwifruit actinidin. Yakhteh Med J.

[16] Spiridonov NA, Konovalov DA, Arkhipov VV (2005). Cytotoxicity of some Russian ethno medicinal plants and plant compounds. Phytother Res.

[17] Auerbach R, Lewis R, Shinners B (2003). Angiogenesis assays: a critical overview. Clin Chem.

[18] Nehls V, Drenckhahn DA (1995). A novel,microcarrier-based in vitro assay for rapid and reliable quantification of three-dimensional cell migration and angiogenesis. Microvasc Res.

[19] Mansouri K, Mostafie A, Rezazadeh D (2016). New function of TSGA10 gene in angiogenesis and tumor metastasis: a response to a challengeable paradox. Hum Mol Genet.

[20] Norooznezhad AH, Norooznezhad F, Ahmadi K (2014). Next target of tranilast: inhibition of corneal neovascularization. Med Hypotheses.

[21] Sauder DN, Dekoven J, Champagne P (2002). Neovastat [AE-941], an inhibitor of angiogenesis: Randomized phase I/II clinical trial results in patients with plaque psoriasis. J Am Acad Dermatol.

[22] Shakiba Y, Mansouri K, Mostafaie A (2007). Anti-angiogenic effect of soybean kunitz trypsin inhibitor on human umbilical vein endothelial cells. Fitoterapia.

[23] Hassan ZM, Feyzi R, Sheikhian A (2005). Low molecular weight fraction of shark cartilage can modulate immune responses and abolish angiogenesis. Int Immunopharmacol.

[24] Mohammadi Motlagh HR, Mansouri K, Shakiba Y (2009). Anti-angiogenic effect of aqueous extract of shallot [Allium ascalonicum] bulbs in rat aorta ring model. Yakhteh Med J.

[25] Keshavarz M, Bidmeshkipoor A, Mostafaie A (2011). Anti tumor activity of Salvia officinalis is due to its anti-angiogenic, anti-migratory and anti-proliferative effects. Cell J (Yakhteh).

[26] Mostafaie A, Mansouri K, Norooznezhad A (2011). Anti-angiogenic activity of ficus carica latex extract on human umbilical vein endothelial cells. Cell J (Yakhteh).

[27] Keshavarz M, Norooznezhad AH, Mansouri K (2010). Cannabinoid (JWH-133) therapy could be effective for treatment of corneal neovascularization. Irn J Med Hypotheses Ideas.

[28] Mansouri K, Mahamed-Khosroushahi L, Rasouli H (2014). Anti-angiogenic/inflammatory behavior of mushroom ganoderma lucidum extract could be effective for treatment of corneal neovascularization: A hypothesis. J Rep Pharm Sci.

[29] Arshadi D, Mansouri K, Khodarahmi R (2014). In vitro anti-angiogenic activity of Persian Shallot (Allium Hirtifolium) extract is mediated through inhibition of endothelial cell proliferation/migration and down-regulation of VEGF and MMP expression. J Rep Pharm Sci.

